# Pelvic organ prolapse surgery and health-related quality of life: a follow-up study

**DOI:** 10.1186/s12905-020-01146-8

**Published:** 2021-01-02

**Authors:** Tadesse Belayneh, Abebaw Gebeyehu, Mulat Adefris, Guri Rortveit, Janne Lillelid Gjerde, Tadesse Awoke Ayele

**Affiliations:** 1 Institute of Public Health, College of Medicine and Health Sciences, University of Gondar, Gondar, Ethiopia; 2 Department of Gynecology and Obstetrics, School of Medicine, University of Gondar, Gondar, Ethiopia; 3 Section for General Practice, Department of Global Public Health and Primary Care, University of Bergen, Bergen, Norway; 4 Research Unit for General Practice, NORCE Norwegian Research Center, Bergen, Norway; 5 Faculty of Health and Social Sciences, Western Norway University of Applied Sciences, Bergen, Norway

**Keywords:** HRQoL, Pelvic reconstructive surgery, Quality of life, Uterine prolapse, Prolapse surgery

## Abstract

**Background:**

Symptomatic prolapse impairs quality of life. Health-related quality of life (HRQoL) is considered an important outcome of pelvic organ prolapse (POP) surgery. However, it is rarely reported, and measures are inadequately used. Thus, studies reporting patient-reported surgical outcomes in low-income contexts are needed. This study aims to evaluate the effect of prolapse surgery on patient HRQoL and determine the predictive factors for change in HRQoL.

**Methods:**

A total of 215 patients who had prolapse stage III or IV were enrolled. Patients underwent vaginal native tissue repair, and their HRQoL was evaluated at baseline, 3 and 6 months postoperatively. Effect of surgery on subjective outcomes were measured using validated Prolapse Quality of Life (P-QoL-20), Prolapse Symptom Score (POP-SS), Body Image in Prolapse (BIPOP), Patient Health Questionnaire (PHQ-9), and Patient Global Index of Improvement (PGI-I) tools. A linear mixed-effect model was used to compare pre- and postoperative P-QoL scores and investigate potential predictors of the changes in P-QoL scores.

**Results:**

In total, 193 (89.7%) patients were eligible for analysis at 3 months, and 185 (86.0%) at 6 months. Participant’s mean age was 49.3 ± 9.4 years. The majority of patients had prolapse stage III (81.9%) and underwent vaginal hysterectomy (55.3%). All domains of P-QoL improved significantly after surgery. Altogether more than 72% of patients reported clinically meaningful improvement in condition-specific quality of life measured with P-QoL-20 at 6 months. An improvement in POP-SS, BIPOP, and the PHQ-9 scores were also observed during both follow-up assessments. At 6 months after surgery, only 2.7% of patients reported the presence of bulge symptoms. A total of 97.8% of patients had reported improvement in comparison to the preoperative state, according to PGI-I. The change in P-QoL score after surgery was associated with the change in POP-SS, PHQ, BIPOP scores and marital status (*p* < 0.001). However, age, type of surgery, and prolapse stage were not associated with the improvement of P-QoL scores.

**Conclusions:**

Surgical repair for prolapse effectively improves patient’s HRQoL, and patient satisfaction is high. The result could be useful for patient counselling on the expected HRQoL outcomes of surgical treatment. Surgical service should be accessible for patients suffering from POP to improve HRQoL.

## Background

Pelvic organ prolapse (POP) occurs when the pelvic floor no longer supports the proper positioning of the pelvic organs, resulting in the descent of organs through the vagina [1]. It is a common gynecologic condition that is strongly associated with childbirth, ageing and the menopause [1]. Women with POP present a variety of symptoms (vaginal, bladder, bowel and sexual) that greatly affect their daily activities and HRQoL [2, 3]. This results in a significant economic burden to the patients and healthcare system [4]. Although most cases are asymptomatic and treated conservatively [5], up to 20% require surgery during their lifetime [6]. In Ethiopia, unlike with Obstetric Fistula, there is no free surgical service for prolapse. However, the Ethiopian Ministry of Health, in collaboration with the United Nations Population Fund and Women and Health Alliance International, regularly organises the “POP surgical campaign” in selected government hospitals to treat symptomatic patients [7]. A recent study in Ethiopia reported that 17% of women had symptomatic POP that require surgical treatment [8].

The primary goal of POP surgery is to provide quality care with meaningful patient impact, i.e. reduce symptoms and improve HRQoL [9]. Previously, however, most studies evaluating surgical success have focused exclusively on the change in anatomical prolapse stage. Patient-reported outcomes (PRO) such as symptom change, satisfaction, and change in the HRQoL are considered equal or more important when comparing the success of various POP surgeries [9, 10]. This is because anatomic criterion did not demonstrate the strongest relationships with the patients’ assessment of overall improvement, treatment success, improvements in symptom bother and HRQoL.

Measurement of HRQoL using validated instruments is increasingly common in POP surgery [11]. However, PRO measures are infrequently used [12], and the instruments are mainly developed for English-speaking populations [13, 14]. Moreover, while most of the studies with patient-reported HRQoL outcome measures compare selected surgical methods in one vaginal compartment prolapse outcome [15], most of the patients need multiple vaginal compartment prolapse repair [16]. Thus, studies reporting surgical outcomes of non-selected patients are needed.

In low and middle-income countries (LMICs) including Ethiopia, the effect of prolapse surgery on HRQoL is rarely reported, and the use of PRO measures is inadequate. Therefore, evidence concerning the impact of POP surgery on HRQoL with validated instruments is needed. This study aims to evaluate the effect of prolapse surgery on patient symptoms and HRQoL and determine the predictive factors for change in HRQoL.

## Methods

A single-group longitudinal study was conducted on those patients admitted to the University of Gondar referral hospital (UoGH) from February 2018 to May 2019. The study period ended in November 2019 with the 6 month follow-up of the last study subjects. The inclusion criteria were age ≥ 18 years, POP stage III or IV based on the Pelvic Organ Prolapse Quantification (POP-Q) system [17], and the ability to communicate in written and/or oral Amharic language. Patients who had underwent abdominal prolapse surgery, had current co-morbidities, or previously undergone POP surgery or hysterectomy were excluded. A total of 224 patients were calculated assuming a paired mean difference of 3, the standard deviation of the differences of 15 [18], an alpha of 0.05, and a 15% possible loss during the follow-up.

### Surgical procedures

The surgical treatment consisted of the correction of all the defects with a vaginal approach. The surgical method was determined by the severity of prolapse with its compartment level; the prolapse-specific symptoms bother; the patient’s general health, activity level and goals; and the surgeon’s preference and capabilities. Anterior and posterior vaginal prolapse were treated with conventional anterior and posterior colporrhaphy. For apical prolapse, either a vaginal hysterectomy (VH) or uterine-preserving procedure—specifically sacrospinous ligament fixation—was performed. All patients underwent a standardised procedure performed by one of five surgeons (two urogynaecologists and three gynaecologists). All defects were treated by native tissue under spinal anaesthesia. At discharge, patients were informed to avoid sexual intercourse and heavy lifting/workload for approximately 3 months and asked to have a follow-up visit at 3 and 6 months.

### Evaluation of HRQoL

The following PRO instruments were administered preoperatively: Prolapse Quality of Life (P-QoL), Pelvic Organ Prolapse Symptom Score (POP-SS), Body Image in Pelvic Organ Prolapse (BIPOP), and Patient Health Questionnaire (PHQ-9). The P-QoL was used to evaluate women’s HRQoL. The P-QoL included 20-items divided into nine domains: general health perception (GHP), prolapse impact (PI), role (RL), physical (PL) and social limitation (SL), personal relationships (PR), emotional disturbances (EMO), sleep/energy disturbances (SE), and severity measures (SM). Each domain is related to a particular aspect of QoL, and scores in each domain range from 0 to 100. A higher score indicates poor QoL in each domain [13]. This study used a validated Amharic version of P-QoL [19]. The Amharic version had three components: physical (PC; including GHP, PI, PL, RL SL, and SM), psychological (PSC; including EMO and SE), and personal relationship (PRC; containing PR) [19]. The POP-SS was used to evaluate the severity of prolapse symptoms. POP-SS includes seven questions and scored from 0 to 28. Higher scores are indicative of more bothersome symptoms [14]. The present study used the Amharic version of POP-SS [20]. A woman’s perception of her body including genital body image and sense of attractiveness was evaluated using BIPOP. BIPOP includes 10-items, and each item uses a 5-point Likert response with lower scores indicating better body image [21]. The English version was translated into Amharic and then back into English to confirm correctness before use. We asked the patients to assess their depressive symptoms using the PHQ questionnaire. The questionnaire contains 9-items, with higher scores indicating more severe depressive symptoms. It has been validated for use in primary care and obstetrics/gynaecology outpatient clinics to diagnose major depressive disorders [22]. The current study utilised the Amharic version of PHQ [23]. We also evaluated patient’s postsurgical goals. Patients were asked to mention the three topmost goals for their planned surgery. The goals included were dropping prolapse, urinary or bowel symptoms, reducing pain, improving body image, activities and social life, intimate relationships, or general health or living happily. The list of goals were adapted from previous work [24].

At baseline, besides the abovementioned instruments, socio-demographic information (age, residence, marital, employment, and educational status), stage of POP and duration of POP symptoms (the number of years from the time POP symptoms first occurred, classified as delayed in need of healthcare if persisting more than a year) were collected using a standardised form. All baseline interviews were administered face-to-face at UoGH by trained female nurses.

### Patient follow-up

Follow-up data were collected at 3 and 6 months postoperatively using similar instruments (P-QoL, POP-SS, BIPOP, and the PHQ) that were administered preoperatively. We also administered a Patient Global Impression of Improvement (PGI-I) questionnaire. The PGI-I is a single item question that asks patients to rate their subjective improvement after urologic and prolapse treatment on a seven-point Likert scale (1 = very much better, 2 = much better, 3 = a little better, 4 = no change, 5 = a little worse, 6 = much worse, or 7 = very much worse) [25]. In this study, patients were classified as improved if they scored 1, 2, or 3 on the PGI-I scale. The English version was translated into Amharic before administration. Patients were also asked whether they would recommend the operation to others with prolapse symptoms.

### Statistical analyses

Each completed instrument was checked visually for completeness before being fed into a computer. Data were summarised using a mean with standard deviation (SD) for continuous evaluation and numbered with percentages for categorical variables. Patient’s baseline characteristics and HRQoL details were analysed by comparing between those who responded to the 6 month follow-up and those who dropped out. The statistical significance was set at *p* < 0.05. The differences in categorical variables between the respondents and drop-outs were tested with the independent-sample *t* test.

The outcome of the prolapse surgery was evaluated subjectively. The subjective cure was defined as no vaginal bulge symptom (an affirmative response to the POP-SS question “Do you have a bulge or something falling out that you can see or feel in the vaginal area?” with any degree of bother greater than “not at all’’) and improvement in P-QoL score after surgery. The primary outcome was a change in P-QoL scores. Linear mixed-effect models were used to test the statistical significance of the difference in the means of outcome variables at different points of time (e.g. 6-month and baseline values). First, P-QoL outcome measurements (PC, PSC, and PRC subscales) were compared over time using a random intercept model assuming time as a fixed effect. Then, the models were fitted with PC, PSC, and PRC scores as dependent variables and time, age, type of surgery, POP duration, marital status, POP-SS, PHQ, and BIPOP scores as covariates (fixed effect). For each model, we reported the fixed effects coefficients (β value) of the independent variable with the associated 95% CI and *P*-value. A model with random intercepts, slopes and an unstructured covariance structure was employed after model comparison with the Akaike information criterion (AIC). The unstructured covariance structure, which accounts for the within-subject correlation, was chosen based on the model fit using AIC. For all analyses, *p* < 0.05 was considered statistically significant, and normality was assessed using the Shapiro–Wilk test. Statistical analyses were performed by STATA, version 14.0.

## Results

### Patient characteristics

A total of 249 patients underwent POP surgery during the study period, and 23 patients with primary vaginal vault POP after the previous hysterectomy were excluded. Of the 226 patients enrolled for primary POP surgery, 11 revoked their consent before the operation, leaving 215 (97.7%) to take part in the baseline interview. The follow-up questionnaires were received from 193 (89.7%) patients at 3 months and 185 (86.0%) at 6 months after the operation. The primary reasons for leaving the follow-up schedule included having declined further participation (n = 8), lost to follow-up for unknown reasons (n = 8), relocation (n = 10), died for reasons unrelated to complication of prolapse treatment (n = 2), or incomplete data (n = 2). There was no difference in the symptoms (POP-SS and PHQ), body image, POP stage, or P-QoL scores between the patients who participated/did not participate in the follow-up (*p* > 0.05, Additional file 1: Table S1).

A large number had had POP for a long time before seeking treatment (median = 5.2; range 1–26 years). Reasons reported as the main barriers to seeking early treatment were lack of money (21.2%), fear of disclosure (15.9%), the perception that POP is incurable (13.6%), fear of treatment outcome (12.1%), lack of accompanying support (8.6%), distance from a health facility (requiring 2 days or more to reach health facility; 6.2%), and lack of transportation (5.0%). Three out of five patients (59.5%) had the decision making power to visit a health care facility when getting sick.

Patients' characteristics are shown in Table 1. The mean age at the time of surgery was 49.3 ± 9.4 (range 35–70) years. Preoperatively, 148 (68.8%), 134 (62.3%), and 72 (33.5%) patients had anterior, central, and posterior descent ≥ III stages, respectively. These defects were associated with variable degrees of loss of support at the other vaginal sites considered, thus 131 patients (60.9%) showed descent in all three compartments, 50 (23.3%) in two compartments, and 34 (15.8%) in only one compartment (18 anterior descent and 16 apical descent).

**Table 1:** Participant’s baseline characteristics at the University of Gondar Hospital, (N = 215)

Characteristic	N (%)^a^
Age (years), mean (SD)	49.3 (9.4)
Pregnancy, mean (SD)	6.5 ± 2.5
Parity, mean (SD)	5.9 (2.6)
Marital status	
Married/cohabiting	188 (87.4)
Not married^b^	27 (12.6)
Educational status	
Illiterate	204 (94.9)
Literate	11 (5.1)
Occupational status	
Farmer	94 (43.7)
Housewife	84 (39.1)
Other^c^	37 (17.2)
Child < 5 years	
Yes	47 (21.9)
No	168 (78.1)
Duration of POP	
≤ 1 year	59 (27.4)
≥ 2 year	156 (72.6)
Prefer uterine preservation if,	
Preservation > hysterectomy	82 (38.1)
Hysterectomy > preservation	116 (54.0)
Hysterectomy = preservation	85 (39.5)
Category of POP stage III and higher^d^	
Cystocele (anterior compartment)	148 (68.8)
Apical prolapse (middle compartment)	134 (62.3)
Rectocele (posterior compartment)	72 (33.5)
Surgical procedures	
Vaginal hysterectomy	119 (55.3)
Sacrospinous fixation	46 (21.4)
Anterior colporrhaphy	154 (71.6)
Posterior colporrhaphy	87 (40.5)

*POP* pelvic organ prolapse, *SD* standard deviation

^a^Unless specifically stated otherwise

^b^Not married: single or divorced or widowed

^c^Other: student, merchant, and jobless

^d^Several patients had prolapse in more than one compartment, and the sum of the percentages may be greater than 100%

Intraoperative and postoperative results were collected. The median operative time for all the surgical interventions was 65 (47–127) minutes, and the median postoperative hospital stay was 2 days (range, 2–4 days). No intraoperative complications such as severe haemorrhaging or rectal, bladder, or ureteric injuries occurred. However, some women developed mild to moderate postoperative complications: seven cases (3.2%) of fever and 11 (5.1%) of urinary tract infection. Late complications were also reported and included two cases (1.1%) of gluteal pain, four cases (2.5%) of dyspareunia, five cases (2.7%) of stress urinary incontinence, six cases (3.2%) of recurrent urinary tract infections, and five cases (2.7%) of constipation.

### Effect of prolapse surgery on patient’s subjective outcomes

**Fig. 1:**
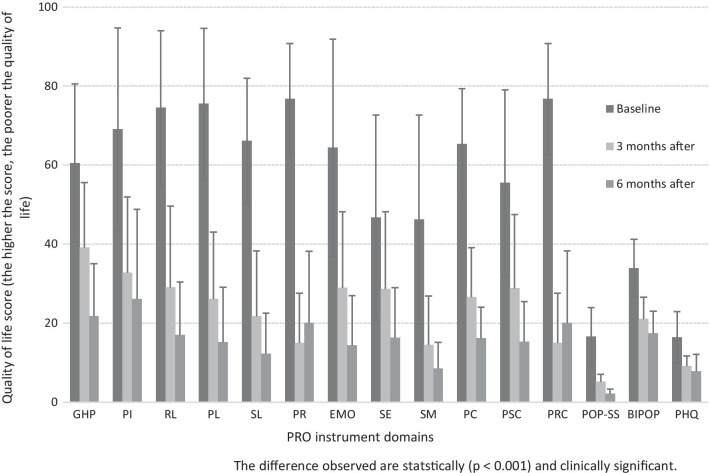
Scores of patient-reported outcome instruments before and after surgical repair of prolapse (Mean ± SD). *GHP* general health perception, *PI* prolapse impact, *RL* role limitation, *PL* physical limitation, *SL* social limitation, *PR* personal relationships, *EMO* emotional disturbances, *SE* sleep/energy, *SM* severity measures, *PC* physical component, *PSC* psychological component, *PRC* personal relationship component, *POP-SS* pelvic organ prolapse symptom score, *BIPOP* body image in pelvic organ prolapse, *PHQ* patient health questionnaire, *PRO* patient-reported outcome

A significant improvement in quality of life was reported throughout the study (Fig. 1 and Table 2). In Fig. 1, the bar chart shows the figures (Mean ± SD) for P-QoL, POP-SS, BIPOP, and PHQ at 3 and 6 months after surgery compared to patients’ scores before surgery. The internal consistency (Cronbach’s alpha) of the P-QoL, POP-SS, BIPOP, and PHQ instrument in this study was 0.92, 0.75, 0.81, and 0.71, respectively.

**Table 2:** Change of health-related quality of life as measured with patient-reported outcome instruments during the 6 months follow-up period at the University of Gondar Hospital, 2019

	Score	Change of score from baseline	
	Mean (95% CI)	Mean (95% CI)	%
P-QoL			
PC			
Baseline	65.3 (63.5, 67.6)		
3 months	26.6 (24.8, 28.3)	− 38.9 (− 41.5, − 36.2)	− 59.6
6 months	16.2 (15.1, 17.3)	− 49.4 (− 51.5, − 47.2)	− 75.2
PSC			
Baseline	55.5 (51.9, 58.7)		
3 months	28.8 (26.1, 31.4)	− 26.8 (− 31.2, − 22.4)	− 48.3
6 months	15.3 (13.8, 16.7)	− 40.1 (− 43.6, − 36.4)	− 72.3
PRC			
Baseline	76.7 (75.9, 80.2)		
3 months	15.0 (13.2, 16.8)	− 54.3 (− 65.0, − 60.1)	− 70.8
6 months	20.0 (17.3, 22.6)	− 58.1 (− 61.8, − 54.3)	− 75.7
POP-SS			
Baseline	16.6 (15.8, 17.0)		
3 months	5.2 (4.9, 5.4)	− 11.1 (− 11.8, − 10.5)	− 66.8
6 months	2.1 (1.9, 2.2)	− 14.3 (− 14.9, − 13.7)	− 86.1
BIPOP			
Baseline	33.9 (33.0, 35.2)		
3 months	21.1 (20.3, 21.8)	− 12.9 (− 13.8, − 12.1)	− 38.1
6 months	17.4 (16.6, 18.2)	− 16.6 (− 17.9, − 15.4)	− 48.9
PHQ			
Baseline	16.4 (15.3, 17.3)		
3 months	9.1 (8.7, 9.5)	− 7.4 (− 8.3, − 6.6)	− 45.1
6 months	7.8 (7.1, 8.4)	− 8.8 (− 9.9, − 7.7)	− 53.6

*BIPOP* body image in pelvic organ prolapse, *HRQoL* health-related quality of life, *POP-SS* pelvic organ prolapse symptom score, *PC* physical component, *PRC* personal relationship component, *PSC* psychological component, *PHQ* patient health questionnaire, *PRO* patient-reported outcome, *UoGH* University of Gondar Hospital

### Prolapse quality of life

Preoperatively, the negative effects on personal relationships (76.7/100 points) and negative impact on physical impairment and roles (75.5 and 74.5/100 points) were those areas of patients’ HRQoL most affected. A significant improvement was reported after the 3 month follow-up for the above-listed domains. However, this improvement was not reproduced in the personal relationship domain score during this follow-up period, as the patient had a worse score (15.0 ± 12.6 to 20.0 ± 18.3, *p* = 0.005, paired *t* test). The 6-month follow-up demonstrated a further significant improvement as compared to the baseline. Similar improvement was also reported in the other P-QoL areas (GHP, SE, and SM) in both follow-ups (baseline vs. 6-month follow-up, out of 100 points in each case: 60.5 vs. 21.8; 46.7 vs. 14.4; 46.2 vs. 8.5; Additional file 1: Table S2).

P-QoL was also higher as measured by PRC, PC, and PSC at baseline. Nevertheless, a marked improvement was observed at a 6 month follow-up (mean change at baseline and 6 months with a 95% CI in each case: − 58.1 (− 61.8, − 54.4), − 49.4 (− 51.5, − 47.2) and − 40.1 (− 43.6, − 36.4), Table 2).

### Prolapse symptoms score

A significant reduction in POP symptoms was detected (Table 2). The POP-SS score decreased at the 3 month follow-up, and the decrease was sustained at the 6-month follow-up (the mean decreased 5.2 and 2.1 points, respectively). At baseline, discomfort/pain that worsens when standing (85.1%), feeling something coming down (82.7%), and feeling heaviness around the lower abdomen (81.4%) were reported. These symptoms were reduced significantly after surgery. A total of 14.0% (n = 166) of patients reported a bothersome bulge symptom at 3 months after surgery, and 97.3% (n = 180) did not report this symptom at 6 months postoperatively (Additional file 1: Table S2).

### Depressive symptoms

A total of PHQ-9 scores > 10 occurred in 42.8% (92/215) of the patients at baseline. After 6 months, the PHQ-9 scores significantly decreased. The mean change PHQ from baseline to 6 months was − 8.8 (95% CI: − 9.9, − 7.7 points, Table 2). The postoperative prevalence of depressive symptoms was 7.0% (13/185), which was six-fold lower compared to baseline. Items representing alterations in doing things, energy, and hope were the most commonly reported items at baseline (Additional file 1: Table S2).

### Body image

Improvement in the BIPOP score was observed, indicating a better BI perception after surgery. Before surgery, 115 patients (53.8%) reported having regular sexual intercourse, while 18 (15.6%) had dyspareunia. Conversely, 6 months after surgery, 159 (85.9%) reported having regular sexual intercourse and 4 (2.5%) had dyspareunia (Additional file 1: Table S2). The BIPOP score (mean of 95%CI) was 33.9 (33.0, 35.2) versus 17.4 (16.6, 18.2) preoperative and at the 6 month follow-up, respectively (*p* < 0.001, Table 2).

### Patient global impression of improvement

**Fig. 2:**
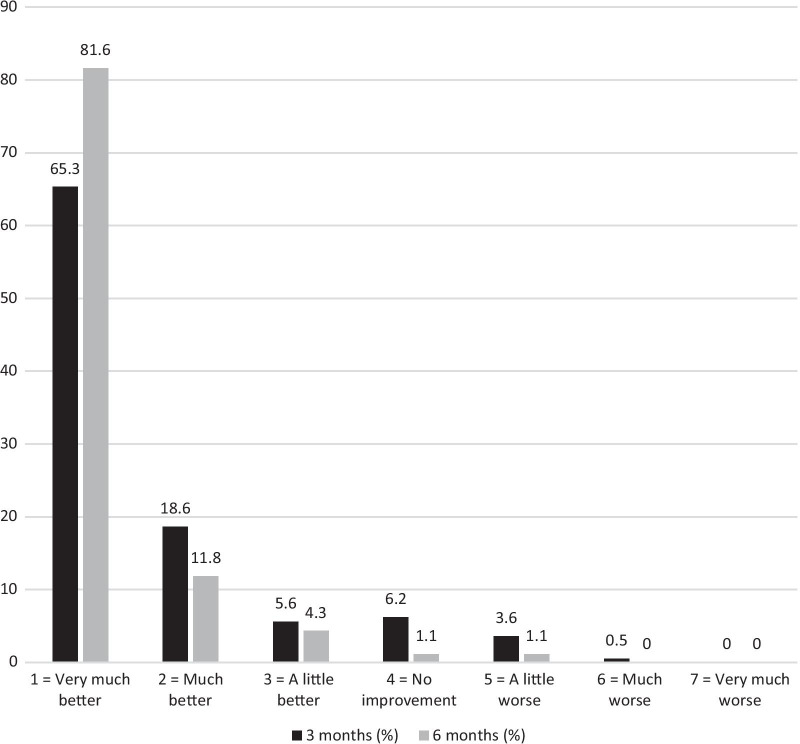
The patient global index of improvement (PGI-I) at 3 months and 6 months after the operation

Response to the surgical treatment measured by the PGI-I is shown in Fig. 2. Altogether, 97.8% of the patients considered their condition to be better, and 1.1% considered it to be worse compared to the preoperative situation at the 6-month follow-up (PGI-I scales 1–3, Fig. 2). At the 6-month follow-up, 171 (92.4%) patients recommended the operation to a close friend suffering from POP.

### Goal attainment

Two hundred and ten women completed the preoperative goal assessment (97.6%). The most common patient goal was to reduce condition-specific symptoms, namely prolapse (186, 88.5%), urinary (174, 82.5%), and bowel symptoms (96, 45.7%), followed by improving intimate relationships (74, 35.2%), activities and social life (66, 31.4%), as well as body image/physical appearance (60, 28.5%) and general health (47, 22.3%). Living happily (74, 35.2%), reduced discomfort/pain (14, 6.7%) and other concerns (22, 10.5%) were also reported. One hundred sixty-six (89.7%) of 185 women achieved their goal of reducing prolapse symptoms (defined as score ≥ 6/10) at 6 month follow-up.

### Predictors of quality of life change during follow-up

Linear Mixed Model (LMM) analysis was performed to examine the longitudinal effects of sociodemographics, POP-SS, BIPOP, and PHQ on P-QoL domains and/or components (Table 3). The change in P-QoL after surgery was associated with the change in POP-SS, PHQ, and BIPOP scores (*p* < 0.001). There was a change in PC and PRC scores overtime for a point change in the POP-SS, PHQ, and BIPOP scores (*p* < 0.001). Being married resulted in a 5.7 point increase in the PRC score (*p* < 0.01). However, age, parity, type of surgery, and prolapse stage were not associated with the improvement of P-QoL scores (Table 3).

**Table 3:** Predictors of change in Prolapse-Quality of Life score using a linear mixed effect model, University of Gondar Hospital, Ethiopia, 2019

Parameter	Estimate	SE	*p* value	95% confidence intervals	
				Lower bound	Upper bound
Results for physical component (PC) of P-QoL					
Intercept	− 66.62	17.34	< 0.001	− 100.63	− 32.62
Marital status	2.55	1.58	0.10	− 0.55	5.65
POP-SS	6.95	1.36	< 0.001	4.28	9.62
PHQ	4.70	1.20	< 0.001	2.33	7.07
BIPOP	3.89	0.60	< 0.001	2.70	5.08
Time	23.06	5.28	< 0.001	12.70	33.41
Results for psychological component (PSC) of P-QoL					
Intercept	− 50.35	26.89	0.06	− 103.07	2.36
Marital status	2.56	2.32	0.27	− 1.99	7.13
POP-SS	5.54	2.11	< 0.01	1.39	9.68
PHQ	4.42	1.86	0.01	0.77	8.07
BIPOP	3.51	0.93	< 0.001	1.69	5.34
Time	14.55	8.06	0.07	− 1.28	30.34
Results for personnel component (PRC) of P-QoL					
Intercept	− 159.38	22.09	< 0.001	− 202.69	− 116.07
Marital status	5.80	1.97	< 0.01	1.93	9.66
POP-SS	12.94	1.70	< 0.001	9.60	16.28
PHQ	5.95	1.53	< 0.01	2.94	8.96
BIPOP	5.69	0.76	< 0.001	4.18	7.19
Time	60.70	6.83	< 0.001	47.29	74.10

*SE* standard error, *BIPOP* body image in pelvic organ prolapse, *P-QoL* prolapse-quality of life, *GHP* general health perception, *PI* prolapse impact, *RL* role limitation, *PL* physical limitation, *SL* social limitation, *PR* personal relationships, *EMO* emotional disturbances, *SE* sleep/energy, *SM* severity measures, *PC* physical component, *PSC* psychological component, *PRC* personal relationship component, *POP-SS* pelvic organ prolapse symptom score, *PHQ* patient health questionnaire, *PRO* patient-reported outcome

## Discussion

This follow-up study revealed an improvement in patients’ prolapse symptoms, body image and HRQoL after repair of POP. Postoperatively, at 6 months after surgery, the majority of patients perceived that their condition was improved (97.8%) and reported significant improvement in QoL (72%) compared with the preoperative situation. Accordingly, patient satisfaction was high.

This adds to the growing body of literature that POP surgery is associated with an improvement in HRQoL. To ensure the quality of surgical outcomes, reliable, valid, and easy-to-use measures of surgical quality and patient impact are needed. The P-QoL is a PRO tool that can measure the impact of surgical interventions on patient’s HRQoL. This study uses locally validated P-QoL to demonstrate an improvement in patient-reported HRQoL after native tissue repair of prolapse in Ethiopia [19]. Thus, our study adds to the evidence that P-QoL can be used as a PRO tool to demonstrate patient impact after surgery in LMIC, specifically in Ethiopia.

Surgical intervention of prolapse can improve HRQoL in women with POP [2]. Our study shows that repair of POP improves prolapse-specific HRQoL at 3 and 6 months after the procedure.

The P-QoL of the study participants had improved even more, and the difference between the preoperative figures remained significant. The improved P-QoL scores compared to baseline were also observed in the PC, PSC, and PRC of the P-QoL instrument after 6 months. One explanation for these findings is the improvement of symptoms, which leads to improvement of the different aspects of the HRQoL. Patients with stage III-IV often report multiple and bothersome symptoms that warranted the risk of POP repairs for those symptoms [26]. In our study, all of the patients were above stage II and had undergone multiple surgical procedures. In reality, prolapse often involves multiple vaginal compartments, and the surgical method is chosen based on clinical judgment as there is no single procedure that improves all prolapse symptoms. Therefore, surgical correction of the underlying problem addresses these concerns, and negative perceptions might be reduced when prolapse symptoms are eliminated. Furthermore, symptom relief and improved QoL are recognised as the determining factors for surgical success. Our findings are in line with previous studies in Western countries showing that surgical treatment improves HRQoL among women suffering from POP [27–29]. Qualitative findings from Ethiopia also reported great benefits in many aspects of life after POP surgery [30]. The average scores for prolapse effects on physical and role activities and personal relationships were quite high at baseline. A previous study carried out in European women also showed similar scores for the same domains [18, 27, 31]. However, daily life for Ethiopian rural women (e.g. the burden of physical activities or work, gender inequality affecting PR, shame, lack of education/knowledge, etc.) is hardly comparable to women living in Europe. The remarkable improvement found in P-QoL domain scores (PC and PRC) after surgery could have a positive influence on formerly impaired HRQoL among those affected. This is similar to a study from Nepal [32], reporting a significant improvement in every aspect of the QoL measured. Our results imply that, in rural settings where nearly all the housework was performed by women alone or with the help of their children, and where women often help out with heavy farming activities [33], accessing surgical services improves overall QoL and enables them to perform daily household and/or outdoor roles like fetching water from distant sources, participating in farming activities and helping care for children under the age of five (21.9% had children under five at the time of surgery).

In the current study, more than nine out of ten patients experienced a symptom-free life 6 months after the surgery. Furthermore, total score of POP-SS was significantly reduced after surgery. Similar results of symptom improvement have been reported elsewhere [34], although the mean decrease of POP-SS scores were higher (3.2) than our study (2.1) after the surgery. This difference might be due to the inclusion of a specific vaginal compartment prolapse. Patients with specific vaginal compartment prolapse may have a greater potential for symptom improvement than those with multicompartment prolapse. Our study result may be helpful for clinicians when they counsel patients about the outcomes of surgical treatment for POP. Furthermore, our observation of improvement may motivate women suffering from POP to seek help.

In our study, a worse body image score was reported preoperatively. However, surgical intervention was effective in improving body image score and reducing dyspareunia 6 months after surgery. Before surgery, 115 patients (53.8%) reported having regular sexual intercourse, and this number increased to 159 (85.9%) at the 6 month follow-up. Only four patients (2.5%) reported dyspareunia. Since the sexual function is strongly correlated with self-perceived BI, the result might indicate indirectly the improvement of sexual function. Similarly, studies also found better BI and sexual satisfaction after surgical intervention [35, 36]. The patient may consider that the genital anatomy altered due to the surgery has a significant impact on their general sense of attractiveness. Moreover, the improvement of BI may be due to the reduction of prolapse symptoms. Evidence shows a strong association between POP symptoms and BI scores [37].

Patients with POP reported worse self-perceived BI, and poor BI is associated with depression and poor psychosocial functioning. Depression is also associated with developing severe POP symptoms, functional impairment, and impaired HRQoL [38]. Thus, depression has a bidirectional relation with QoL in that depression leads to poor QoL and vice versa. We found that surgery leads to a dramatic improvement not only in condition-specific QoL, prolapse symptoms, and body image but also in depressive symptoms 6 months after surgery. This is in line with previous studies showing that surgical treatment improves depressive symptoms among patients suffering from POP [22, 32].

Our study also showed a higher patient satisfaction at the 6 month follow-up. When patient were evaluated using goal attainment and PGI-I score, 98% were satisfied. Moreover, 92% would recommend the surgery to a close friend. This result was better than previous follow-up study of women undergoing either vaginal or abdominal prolapse surgery (72.5% were satisfied with the surgery and 89.7% would recommend the treatment to a friend) [39].

In the present study, the change in the POP-SS, PHQ, and BIPOP score was found to be associated with a change in P-QoL score after POP surgery. However, this follow-up study did not show a significant difference in the P-QoL score among the age group, parity, and stage of POP. A similar result in the age group was also reported elsewhere [40].

Marital status had a significant association with the change in PRC domain score. Those who were married had a greater improvement in HRQoL score than counterparts. This finding is supported by a qualitative study in Ethiopia, which reported that women who lived alone experienced poor improvement in their lives after surgery. For these women, life continued to be a struggle [30]. They also found that avoidance of returning to heavy chores shortly after surgery depended substantially on the support from their family and community members, and proved difficult for those living alone [30]. This might be because the probability of receiving social or relative support is better for those who live in marital bonds.

## Strengths and limitations

To our knowledge, this is the first follow-up study of prolapse surgery and HRQoL that has been published so far in Ethiopia. The major strength of this study is that we evaluated the outcome of surgery using several validated PRO instruments. The use of multiple outcome measures increases the reliability of the study results and allows for comparison with other studies. Furthermore, the availability of these instruments enabled us to evaluate women’s symptoms, HRQoL, and BI in a local context.

There are, however, also some limitations to our work. We have not seen the long-term effect of POP surgery on HRQoL outcomes. Although we reported the 6 month follow-up data, in POP surgery, this is considered relatively short. A long-term follow-up is needed to draw firm conclusions regarding HRQoL. Furthermore, anatomical success rates were not assessed. We do not consider this a major limitation because the post-operative absence of vaginal bulge symptoms significantly correlates with the patient’s assessment of overall improvement, while anatomical success alone does not [9]. To improve the generalisability of our results, we included all surgical pelvic reconstructive surgery methods in all vaginal compartments, and there were large differences in the surgical approaches. This may be considered another limitation, but on the other hand, it does reflect the real-life clinical setting. It is not possible to evaluate if the anterior, posterior, or apical reconstruction surgeries cause a significant improvement in patients’ symptoms and QoL since concomitant or later surgeries in the pelvic area were performed. Furthermore, the study was limited to a single-centre, which might not represent the HRQoL of the patient with POP in Ethiopia. This limits the broader applications of the findings or external validity of the study. However, the involvement of several independent surgeons, the use of standardised operative techniques, and validated outcome measures make our findings generalisable. The single-centre also means that the applied surgical technique was nearly identical for every patient, thus the outcome could be compared. Another limitation is that although we have no reason to doubt the truthfulness of the responses given from respondents, it is conceivable that patients may have withheld less socially desirable responses. The free surgical services received may also affect a patient’s willingness to report a negative outcome. Furthermore, the patient survey based on questions about their QoL, body image, and depressive symptoms are limited as the statements were obtained from the PRO instrument assessment. The final limitation concerns losses to follow-up. Given that subjects who were followed-up were not statistically different from those lost to follow-up, the results were not substantially affected and may be generalisable to the entire surgical population in the Dabat district. A significant bias may occur even with a small proportion of patients lost to follow-up, and more than 20% poses a serious threat to validity in general [41].

## Conclusions

In conclusion, our results show that native tissue repair of POP effectively improves patient’s symptoms, body image and HRQoL, and patient satisfaction is high. More than nine out of ten patients reported better conditions compared to the preoperative situation, and approximately seven out of ten patients achieved significantly better P-QoL over a 6 month follow-up. These results could be used in patient counselling to determine whether to undergo surgical treatment for POP and monitor the patient-centred effect of POP surgery. Access to surgical services for disadvantaged patients may be important to improve HRQoL, and long-term studies evaluating anatomical and functional outcomes of prolapse surgery are also recommended.

## Supplementary Information

**Additional file 1.** Health Related Quality of Life scores among follow-up and lost to follow-up participants.

## Data Availability

The datasets used and/or analysed during the current study are available from the corresponding author upon reasonable request.

## Abbreviations

BIPOP: Body image in pelvic organ prolapse
HIC: High-income countries
HRQoL: Health-related quality of life
LMICs: Low and middle-income countries
POP: Pelvic organ prolapse
POP-SS: Pelvic organ prolapse symptom score
POP-Q: Pelvic organ prolapse quantification
PHQ: Patient Health Questionnaire
P-QoL: Prolapse quality of life
PROMs: Patient-reported outcome measures
