# Supplementary material for: Pelvic organ prolapse surgery and health-related quality of life: a follow-up study
Source: BMC Womens Health. 2021 Jan 2;21:4. doi: 10.1186/s12905-020-01146-8 (PMC7778798; doi:10.1186/s12905-020-01146-8)
Supplement: Supplementary file 1 — Additional file 1. Health Related Quality of Life scores among follow-up and lost to follow-up participants. [file 12905_2020_1146_MOESM1_ESM.docx]

**Supplementary Materials**

**Table S1**. Comparison of baseline socio-demographic and HRQoL scores between those who completed the follow-up and those who had lost to follow-up at UoGH, 2019.

| Characteristics | Follow-up  (*n* = 185) | Lost to follow-up  (*n* = 30) | P-value^$^ |
| --- | --- | --- | --- |
| Age, years (mean ± SD) | 49.2±9.2 | 49.8±10.7 | 0.77 |
| Pregnancies (mean ± SD) | 6.6±2.5 | 6.1±2.7 | 0.39 |
| Parity (mean ± SD) | 5.9±2.5 | 5.8±2.8 | 0.80 |
| Marital status, n (%) |  |  | 0.08 |
| Married/cohabiting | 158 (85.4) | 18 (100.0) |  |
| Not married* | 27 (14.6) | 0 (0) |  |
| Education status, n (%) |  |  | 0.35 |
| Illiterate | 176 (95.1) | 28 (93.3) |  |
| Literate | 9 (4.9) | 2 (6.7) |  |
| POP stage, n (%) |  |  | 0.64 |
| Stage III | 151 (81.6) | 25 (83.3) |  |
| Stage IV | 34 (18.4) | 5 (16.7) |  |
| P-QoL domain scores |  |  |  |
| PC (mean ± SD) | 65.5±14.2 | 64.3±12.9 | 0.67 |
| PSC (mean ± SD) | 55.3±23.7 | 56.9±22.3 | 0.71 |
| PRC (mean ± SD) | 76.4±13.9 | 78.8±15.1 | 0.40 |
| POP-SS score (mean ± SD) | 16.4±4.2 | 17.9±4.7 | 0.06 |
| PHQ score (mean ± SD) | 16.7±6.3 | 14.5±7.2 | 0.09 |
| BIPOP score (mean ± SD) | 34.2±7.4 | 32.2±6.6 | 0.16 |

^$^ Independent sample t-test.

* Not married: single or divorced or widowed.

Abbreviations: SD: standard deviation, PC: Physical component, PSC: psychological component, PRC: personal relationship component.

**Table S2**. Change of HRQoL as measured with PRO instruments during the 6 months follow-up period at UoGH, 2019.

| Variables | Baseline^a^ | | At 3 months | | At 6 months | |  |
| --- | --- | --- | --- | --- | --- | --- | --- |
|  | Mean±SD | 95% CI | Mean±SD | 95% CI | Mean±SD | 95% CI | P-value^b^ |
| **HRQoL** | |  |  |  |  |  |  |
| P-QoL domains | |  |  |  |  |  |  |
| GHP | 60.5 ± 20.1 | 57.1, 63.1 | 39.2 ± 16.4 | 35.9, 44.5 | 21.8 ± 13.3 | 19.9, 23.7 | < 0.001^#^ |
| PI | 69.1 ± 25.6 | 66.2, 73.9 | 32.8 ± 19.1 | 29.4, 37.2 | 26.1 ± 22.7 | 22.8, 29.4 | < 0.001 |
| RL | 74.5 ± 19.5 | 71.1, 76.7 | 29.0 ± 20.6 | 25.7, 34.1 | 17.0 ± 13.4 | 15.0, 18.9 | < 0.001 |
| PL | 75.5 ± 19.1 | 72.5, 78.1 | 26.1 ± 16.9 | 24.4, 32.8 | 15.2 ± 13.9 | 13.2, 17.2 | < 0.001 |
| SL | 66.1 ± 15.9 | 64.2, 68.8 | 21.8 ± 16.5 | 23.9, 32.4 | 12.2 ± 10.3 | 10.7, 13.7 | < 0.001 |
| PR | 76.7 ± 14.1 | 75.9, 80.2 | 15.0 ± 12.6 | 23.8, 32.6 | 20.0 ± 18.2 | 17.3, 22.6 | < 0.001 |
| EMO | 64.4 ± 27.5 | 59.9, 67.7 | 28.9 ± 19.3 | 25.4, 33.4 | 14.4 ± 12.6 | 12.5, 16.2 | < 0.001 |
| SE | 46.7 ± 26.0 | 42.8, 50.6 | 28.6 ± 19.6 | 25.2, 32.8 | 16.3 ± 12.7 | 14.4, 18.1 | < 0.001 |
| SM | 46.2 ± 26.5 | 42.4, 50.2 | 14.5 ± 12.4 | 15.2, 22.6 | 8.5 ± 6.6 | 7.5, 9.5 | < 0.001 |
| P-QoL components | |  |  |  |  |  |  |
| PC | 65.3 ± 14.0 | 63.5, 67.6 | 26.6 ± 12.5 | 24.8, 28.3 | 16.2 ± 7.8 | 15.1, 17.3 | < 0.001 |
| PSC | 55.5 ± 23.5 | 51.9, 58.7 | 28.8 ± 18.7 | 26.1, 31.4 | 15.3 ± 10.1 | 13.8, 16.7 | < 0.001 |
| PRC | 76.7 ± 14.1 | 75.9, 80.2 | 15.0 ± 12.6 | 13.2, 16.8 | 20.0 ± 18.3 | 17.3, 22.6 | < 0.001 |
| **Sexual function** | |  |  |  |  |  |  |
| Sexual activity (%) | 115 (53.8) |  | NA* |  | 159 (85.9) |  | 0.01 |
| BIPOP | 33.9 ± 7.3 | 33.0, 35.2 | 21.1 ± 5.4 | 20.3, 21.8 | 17.4 ± 5.6 | 16.6, 18.2 | < 0.001^#^ |
| **POP symptoms** | |  |  |  |  |  |  |
| Vaginal bulge (%) | 82.7 |  | 14.0 |  | 2.7 |  | < 0.001 |
| POP-SS | 16.6 ± 4.3 | 15.8, 17.0 | 5.2 ± 1.8 | 4.9, 5.4 | 2.1 ± 1.2 | 1.9, 2.2 | < 0.001 |
| **Depressive symptoms** | |  |  |  |  |  |  |
| PHQ | 16.4 ± 6.5 | 15.3, 17.3 | 9.1 ± 2.6 | 8.7, 9.5 | 7.8 ± 4.3 | 7.1, 8.4 | < 0.001^#^ |
| PHQ > 9 (*n,* %) | 92 (42.8) |  | 29 (15.0) |  | 13 (7.0) |  | < 0.001 |

^a^ Baseline scores as reference.
^b^P-value is calculated using a linear mixed-effect model unless specified. P-value is similar for changes from baseline to 3 and 6 months.

^#^ Wilcoxon signed-rank test.

The higher the recorded score of P-QoL, POP-SS, and BIPOP, the lower the QoL, the severity of POP symptoms, and body image perception.

*Sexual activity was not advised until 3 months after surgery.
